# Systemic administration of β-glucan induces immune training in microglia

**DOI:** 10.1186/s12974-021-02103-4

**Published:** 2021-02-22

**Authors:** Yang Heng, Xiaoming Zhang, Malte Borggrewe, Hilmar R. J. van Weering, Maaike L. Brummer, Tjalling W. Nijboer, Leo A. B. Joosten, Mihai G. Netea, Erik W. G. M. Boddeke, Jon D. Laman, Bart J. L. Eggen

**Affiliations:** 1 Department of Biomedical Sciences of Cells & Systems, Section Molecular Neurobiology, University of Groningen, University Medical Center Groningen, Antonius Deusinglaan 1, 9713 AV Groningen, The Netherlands; 2 Department of Internal Medicine and Radboud Center for Infectious Diseases (RCI), Radboud University Medical Center, Nijmegen, The Netherlands; 3 Department for Genomics & Immunoregulation, Life and Medical Sciences Institute (LIMES), University of Bonn, Bonn, Germany; 4 Center for Healthy Ageing, Department of Cellular and Molecular Medicine, University of Copenhagen, Blegdamsvej 3B, 2200 Copenhagen, Denmark

**Keywords:** microglia, innate immune memory, training, tolerance, β-glucan, lipopolysaccharide, morphology, cytokine

## Abstract

**Background:**

An innate immune memory response can manifest in two ways: immune training and immune tolerance, which refers to an enhanced or suppressed immune response to a second challenge, respectively. Exposing monocytes to moderate-to-high amounts of bacterial lipopolysaccharide (LPS) induces immune tolerance, whereas fungal β-glucan (BG) induces immune training. In microglia, it has been shown that different LPS inocula *in vivo* can induce either immune training or tolerance*.* Few studies focused on impact of BG on microglia and were only performed *in vitro.* The aim of the current study was to determine whether BG activates and induces immune memory in microglia upon peripheral administration *in vivo.*

**Methods:**

Two experimental designs were used. In the acute design, mice received an intraperitoneal (i.p.) injection with PBS, 1 mg/kg LPS or 20 mg/kg BG and were terminated after 3 h, 1 or 2 days. In the preconditioning design, animals were first challenged i.p. with PBS, 1 mg/kg LPS or 20 mg/kg BG. After 2, 7 or 14 days, mice received a second injection with PBS or 1 mg/kg LPS and were sacrificed 3 h later. Microglia were isolated by fluorescence-activated cell sorting, and cytokine gene expression levels were determined. In addition, a self-developed program was used to analyze microglia morphological changes. Cytokine concentrations in serum were determined by a cytokine array.

**Results:**

Microglia exhibited a classical inflammatory response to LPS, showing significant upregulation of *Tnf*, *Il6, Il1β*, *Ccl2*, *Ccl3* and *Csf1* expression, three h after injection, and obvious morphological changes 1 and 2 days after injection. With an interval of 2 days between two challenges, both BG and LPS induced immune training in microglia. The training effect of LPS changed into immune tolerance after a 7-day interval between 2 LPS challenges. Preconditioning with BG and LPS resulted in increased morphological changes in microglia in response to a systemic LPS challenge compared to naïve microglia.

**Conclusions:**

Our results demonstrate that preconditioning with BG and LPS both induced immune training of microglia at two days after the first challenge. However, with an interval of 7 days between the first and second challenge, LPS-preconditioning resulted in immune tolerance in microglia.

**Supplementary Information:**

The online version contains supplementary material available at 10.1186/s12974-021-02103-4.

## Background

In recent years, it has become evident that in partial analogy to adaptive immune cells, innate immune cells, like monocytes and macrophages, also can acquire immune memory. This immunological memory has been referred to as innate immune memory [1, 2]. Innate immune memory can manifest in two different ways, immune training and immune tolerance, which means an enhanced or suppressed immune response towards a secondary challenge. In monocytes, immune training and tolerance are two distinct functional fates induced by specific microbial ligands and underpinned by epigenetic reprogramming [3–5].

Lipopolysaccharide (LPS) and β-glucan (BG) are two commonly used ligands to induce immune tolerance and training in monocytes/macrophages, respectively. LPS is a cell wall component of Gram-negative bacteria and a potent trigger of inflammatory responses in monocytes/macrophages by activating Toll-like receptor (TLR) 4 [6]. After the first LPS challenge, animals or cultured monocytes/macrophages become refractory to a second challenge of LPS or other inflammatory stimuli, a phenomenon called LPS tolerance [7]. In monocytes/macrophages, LPS tolerance requires hypoxia-inducible factor-1α (HIF1α) [8], and is mediated by epigenetic modifications through transcription factors RelB and ATF7 [9–11]. LPS tolerance resembles the immune paralysis observed in sepsis patients or other non-infectious systemic inflammatory response syndrome [12–14]. The first LPS challenge can reduce a second LPS-induced febrile response, metabolic changes and lethality from experimental models of septic shock [7]. In addition, LPS preconditioning was shown to be protective against subsequent cerebral ischemic injury by the induction of neuroprotective spleen monocytes, which mobilize to the brain and meninges, suppressing post-ischemic inflammation [15, 16]. Thus, LPS tolerance in general is viewed as a protective adaption to a second challenge. Nevertheless, LPS preconditioning also sometimes induces detrimental effects. Mice preconditioned with a super-low dose (5 ng/kg body weight) of LPS displayed exacerbated sepsis-induced tissue damage, bacterial load in circulation, and mortality [17].

BG is the most abundant fungal cell wall polysaccharide and a promising biological response modifier for the treatment of cancer and infectious diseases [18, 19]. BGs share a common structure consisting of a backbone of β-(1,3)-linked glucose residues, but their length and branching structure vary considerably. BGs isolated from different sources with different structural complexity have different immunomodulatory properties [20, 21]. BG preconditioning can induce immune training in human and mouse monocytes *in vitro* and protects mice against an otherwise lethal infection with *C. albicans* [22]. The *in vitro* training is characterized by epigenetic programming and a metabolic shift to aerobic glycolysis, which requires the BG receptor Dectin-1 (CLEC7A), the non-canonical Raf-1 pathway and Akt-mTOR-HIF-1α pathways [3, 22, 23]. BG also induces immune training in the periphery *in vivo*, indicated by enhanced serum cytokine concentrations in response to a secondary LPS stimulation [24].

Microglia, the innate immune cells of the central nervous system (CNS), can adopt diverse phenotypes and functions in health and disease [25]. The capacity of microglia to develop innate immune memory has recently been demonstrated [26]. In our previous study, we have shown that LPS preconditioning induces immune tolerance in microglia, characterized by suppressed *Tnf*, *Il1b* and *Il6* gene expression in response to a second LPS challenge. This phenomenon was observed in a microglial cell line, in primary microglia, and *in vivo* [27]. For *in vivo* experiments, we showed that a single intraperitoneal (i.p.) injection of LPS induced immune tolerance in microglia for 1, 4 and even 32 weeks after injection [27]. Wendeln et al. used a different LPS challenge paradigm, and showed that a single LPS injection induced immune training whereas four consecutive daily intraperitoneal (i.p.) LPS injections induced microglia tolerance in mice [28]. Studies on how different LPS treatment schedules and inocula induce different types of microglia immune memory were extensively reviewed by Neher and Cunningham [26].

Compared to LPS, relatively little is known about the effects of BG on microglia. In primary microglia *in vitro*, BG induces phagocytosis and radical oxygen species (ROS) production via the Dectin-1 without inducing significant cytokine production [29, 30]. Co-stimulation of primary microglia with BG suppresses TLR2- (by Pam3Csk4) and TLR4-mediated (by LPS) activation of nuclear factor-κB (NF-κB) [31]. Moreover, in BV-2 cells, pre-treatment with BG reduces LPS-induced *Tnf* expression and NF-κB activation [32]. However, to date, no studies have studied whether BG activates microglia and induces immune memory *in vivo*. In this study, we report for the first time that systemic administration of BG activates microglia *in vivo*, and that BG preconditioning induces immune training in microglia.

## Methods

### BV-2 cell culture and luciferase assays

BV-2 cells were cultured in Dulbecco’s Modified Eagle Medium (DMEM, Lonza, BE12-707F) supplemented with 10% FCS (Bovogen Biologicals, Keilor East, Australia) and 1% pen/strep (GE Healthcare, Little Chalfont, UK) at 37°C in a humidified atmosphere at 5% CO_2_. For luciferase assays, BV-2 cells with a luciferase reporter gene driven by consensus NF-κB binding sites were used. The high sensitivity luminescence reporter gene assay system kit (Steadylite plus, PerkinElmer) was used to measure the luciferase activity and the signal was recorded in a Luminometer. The BV-2 NF-κB luciferase reporter cell line was generated by transduction of BV-2 cells with lentiviral Cignal™ Lenti Reporters (Luc) following the manufacturer's protocol (Qiagen, CLS-013L). Afterwards, the transduced BV-2 cells were seeded in 6 well plates with 4 mg/mL puromycin in the culture medium. The single colonies were selected and expanded. The activation of NF-κB by LPS was analyzed as previously described [33, 34].

### Primary microglia culture

Primary neonatal microglia were isolated from brains of postnatal day 0-2 C57Bl/6J mouse pups as described previously [27]. Briefly, the brains were minced after removing the cerebellum and meninges, followed by 25-35 min 0.25% trypsin dissociation. The tissue was then triturated using glass pipettes. Cells were centrifuged and resuspended in microglia medium incubated at 37°C with 5% CO_2_. The microglia medium is DMEM supplemented with 10% FCS (Gibco, 10500-064), 1 mM sodium pyruvate (Gibco, 11360-070) and 1% penicillin/streptomycin (GE Healthcare, P11-010). When confluent, the medium was refreshed by 10 mL microglia medium and 5 mL LCCM (L929 cell line conditioned medium, DMEM with 10% FCS and 1% penicillin/streptomycin). Three to four days later, microglia were collected by orbital shaking at 37°C and 150 rpm/min for 1 h. Afterwards, the microglia-enriched cell suspension was centrifuged. The supernatant was filtered (20 μm filter) and used as conditioned medium. The microglia were seeded and cultured in the microglia culture medium (fresh microglia medium containing 50% conditioned medium collected after shake off). For stimulation, one day after microglia were seeded, the medium was refreshed with microglia culture medium containing PBS, LPS (100 ng/mL) or β-glucan (10 μg/mL). At 3 h after the stimulation, cells were washed with PBS and total RNA was isolated using TRIzol™ Reagent (Thermo Fisher Scientific, 15596026).

### Animals

All the animal work was performed in the Central Animal Facility (CDP) approved by Animal Care and Use Committee (DEC) of the University of Groningen with protocol (IvD 15360-03-002). C57Bl/6J mice (male, 8-10 weeks, 25-30 grams) were purchased from Envigo (Harlan, the Netherlands). Mice were individually housed under a 12/12 h light/dark cycle (8 p.m. lights off, 8 a.m. lights on) with *ad libitum* access to food and water. Two experimental designs were used (acute and preconditioning). For the acute design, animals were challenged once via i.p. injection with LPS (Sigma-Aldrich, *E. coli*, O111:B4, L4391, 1 mg/kg body weight) or BG (Sigma-Aldrich, *S. cerevisiae*, G5011-25MG, 20 mg/kg body weight) dissolved in Dulbecco’s Phosphate Buffered Saline (PBS). The control mice received a respective volume of PBS. The animals were terminated 3 h, 1 day or 2 days after injections. For the preconditioning design, mice were challenged on day 0 with PBS, 1 mg/kg LPS or 20 mg/kg BG. On day 2, 7 or 14, the mice received a secondary injection of PBS or 1 mg/kg LPS and were sacrificed 3 h later. The mice were put under deep anesthesia and blood was collected in MiniCollect serum separator tubes (Greiner Bio-one, 450472). Next, mice were perfused with saline, brains were isolated and divided into two hemispheres, used for microglia isolation and immunohistochemistry respectively.

### Microglia isolation

Microglia were isolated from adult mouse brain using standardized procedures as described before [35]. Briefly, the brain hemispheres were mechanically dissociated using a tissue homogenizer. The homogenized brain samples were passed through a 70 μM cell strainer to obtain single cell suspensions which were centrifuged at 220 g for 10 min at 4 °C. The supernatants were removed and the pellets were resuspended in 24% Percoll (GE Healthcare, 17-0891-01) gradient buffer with 3 mL PBS on top. Myelin was removed by centrifuging at 950 g for 20 min at 4 °C. The resulting cell pellets were incubated with an antibody solution, containing Cd11b-PE (eBioscience, 12-0112-82), Cd45-FITC (eBioscience, 11-0451-85) and Ly6C-APC (Biolegend, 128015). Microglia were isolated using fluorescence-activated cell sorting (FACS) as DAPI^neg^ Cd11b^high^ Cd45^int^ Ly6C^neg^ events. The cells were collected and centrifuged at 600 g for 10 min. Subsequently, the pellets were lysed in RLT-Plus buffer from RNeasy® Micro Kit (Qiagen, 74004) and stored at -80 °C for RNA isolation.

### RNA isolation and Quantitative RT-PCR (qPCR)

The microglia RNA was isolated using the RNeasy® Micro Kit following the manufacturer’s instructions. The isolated RNA was mixed with 1 μL random primers (0.5 μg/μL, Invitrogen, 48190011) and water to 10 μL. Samples were incubated at 65°C for 15 min and kept on ice. Thereafter, 8 U/μL M-MuLV reverse transcriptase (Thermo Scientific, EP0442), 0.8 U/μL Ribolock RNase inhibitor (Thermo Scientific, EO0382), 0.5 mM dNTP-mix (Thermo Scientific, R0192) and reverse transcriptase buffer were added and incubated on a thermal cycler at 42 °C for 1h, at 70 °C for 10 min and finally at 4 °C. The resulting cDNA was used for qPCR reactions. The PCR reaction mixture contained 5 μL diluted cDNA template from microglia samples, 5.5 μL iTaq™ Universal SYBR® Green Supermix (Bio-Rad, 1725125), 0.3 μL H_2_O and 0.2 μL 10 μM primer mix. Each sample was run with 2 to 3 technical replicates. Quantitative PCR reactions were performed using the QuantStudio 7 Real-Time PCR system (Thermo Scientific) and *Gapdh* or *Hrpt1* was used as a reference gene. Primer sequences are provided in Additional file 1.

### Cytokine antibody array

A commercial cytokine antibody array (RayBiotech, AAM-CYT-3-8) was used to determine cytokine levels in the serum. Sera from replicates per experimental group were pooled together and diluted 4x. The arrays were blocked using blocking buffer for 30 min at room temperature (RT). After aspirating the buffer, the diluted samples were added to the arrays and left to incubate overnight at 4°C. The arrays were washed and incubated with a biotinylated antibody cocktail for 2 h at RT. After a second wash, HRP-streptavidin was added to the arrays, followed by incubation for another 2 h at RT. After a final wash, excess wash buffer on the membranes was removed and detection buffer was added. The cytokine images were taken using the Odyssey Fc imaging system (LI-COR biosciences). Densitometric analysis of the immunoreactivity of each cytokine was performed using image analysis software (QuantityOne). To avoid the background noise, cytokines or chemokines of which density level is below the negative control on any membranes were excluded.

### Immunohistochemistry

Brain hemispheres were fixed for 48 h in 4% paraformaldehyde (PFA) at 4°C. After dehydration in 30% sucrose, the brain samples were embedded with O.C.T. compound (Sakura Finetek, 4583) and stored at -80°C. Sixteen μm thick brain sections were prepared with the cryostat. The sections were dried in the desiccator for 30 min, followed by fixation with 4% PFA in PBS for 20 min. After washing thrice with 1x PBS (identical for all subsequent washing steps), antigen retrieval (AR) was performed using 10 mM sodium citrate, pH 6.0. After washing, the sections were incubated in PBS with 1% hydrogen peroxide (H_2_O_2_) to block endogenous peroxidase. The sections were washed and blocked using 5% normal donkey serum (NDS; Jackson Immuno Research) in PBS^+^ (PBS with 0.3% Triton X-100). The sections were incubated with the primary rabbit-α-ionized calcium-binding adapter molecule 1 (Iba1) antibody (1:1000; Wako, 019-19741) overnight at 4 °C. The following day, the slides were washed and incubated with the biotinylated secondary donkey-α-rabbit IgG antibody (1:400; Jackson Immuno Research, 711-065-152) for 1 h. After washing, the sections were incubated with ABC solution (VECTASTAIN® ABC Kit, Vector Laboratories, PK-6100) for 30 min. The sections were washed, stained using 0.04% 3,3’-Diaminobenzidine (DAB) and 0.01% H_2_O_2_ for 8 min and subsequently dehydrated using increasing ethanol concentrations. The slides were left to air dry for 30 min, mounted with coverslips using DePex (Serva) and stored at RT.

### Morphometric analysis of microglia

All the slides were scanned with the NanoZoomer Digital Pathology system (Hamamatsu Photonics, K.K., Japan) with 40X objective resulting in NDPI files. A pipeline was developed to computationally analyze a panel of 23 morphological features in microglia (Van Weering et al., in prep.). Briefly, single-cell images of Iba1-positive cells were first extracted from the whole slide scans. Next, the single-cell images were pre-processed to cell silhouette images by semi-automated thresholding. Subsequently, the cell silhouettes were converted to cell skeleton images by repeated thinning and pruning of the branch areas. In the cell skeleton, branch endings (end nodes), branch crossings (junctions) and all branchpoints emanating from the cell soma (start nodes) were tagged respectively to allow node quantification. Both cell silhouette and cell skeleton images served as input for fully automated morphometric analysis. The outputs of the pipeline included Sholl analysis [36], and 23 morphometric features per cell. A specified list of morphometric features and a detailed description of the pipeline were described elsewhere (Van Weering et al., in prep.).

### Clustering of microglia based on morphometric features

To identify groups of microglia with similar morphology, a non-supervised clustering approach was applied as described before (Van Weering et al., in prep.). In brief, after normalization and scaling of all morphometric features, a principal component analysis (PCA) was applied to reduce dimensionality and redundancy in the dataset. Subsequently, hierarchical clustering based on Ward’s method was performed on the top-contributing principal components (PCs) with an eigenvalue > 1. For example, for the acute design in the cortex region, the top five PCs (PC1-5) with an eigenvalue > 1 were retained for clustering (Additional file 2a), resulting in 6 clusters of microglia with distinct morphological properties (Additional file 2b). The morphometric properties of each cluster are depicted in Additional file 2c.

### Statistical analysis

Statistical comparisons were performed by one-way ANOVA, followed by Bonferroni multiple comparison. For the morphometric data, type II Wald chi-square test for linear mixed models was used to compare experimental groups. For all statistical tests, the significance level was set to *p* < 0.05.

## Results

### BG activates microglia without inducing significant cytokine expression *in vivo*

First, to determine whether BG could activate microglia, its effect on primary microglia and a microglia BV-2 cell line carrying a NF-κB luciferase reporter gene was analyzed. We first investigated the time course of LPS- and BG-induced NF-κB activation by measuring luciferase activity. Stimulation of BV-2 luciferase cells with 100 ng/mL LPS resulted in significantly increased luciferase activity after 30 min which peaked around 4 h (Additional file 3a). 10 μg/mL BG activated NF-κB signaling within 2 h and peaked around 6 h after stimulation (Additional file 3a). In addition, NF-κB luciferase reporter activity was induced by LPS or BG in a concentration-dependent manner (Additional file 3b). Next, the effect of BG on primary microglia cells was determined. Primary microglia were stimulated with LPS or BG, and after 3 hr, mRNA levels were quantified using RT-qPCR. Both LPS (100 ng/mL) and BG (10 μg/mL) significantly induced *Tnf, Il6, Il1β, Ccl3* and *Csf1* expression in primary microglia (Additional file 3c).

**Fig. 1.**
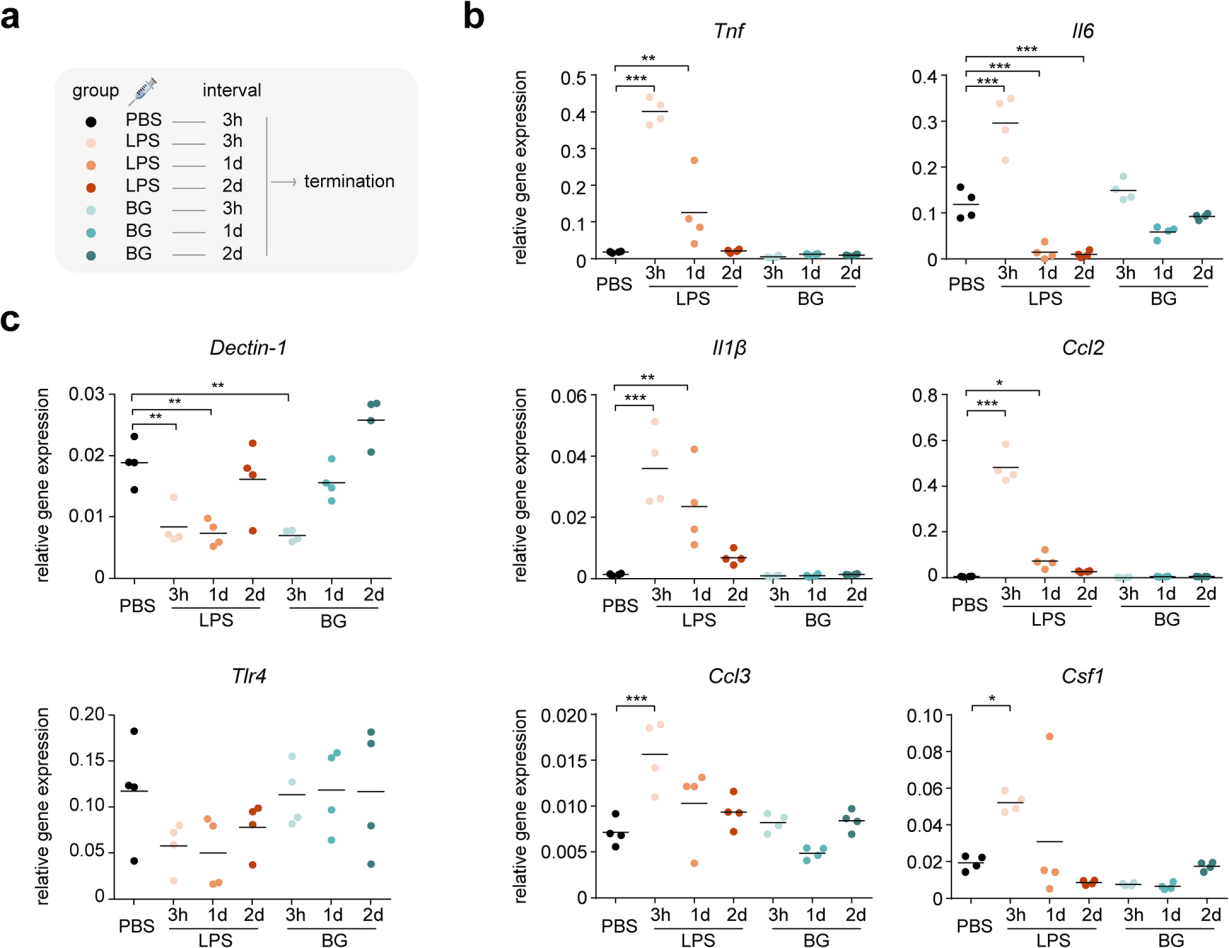
BG activates microglia without inducing significant cytokine gene expression *in vivo*. **a** Acute stimulation study design. Mice received a single challenge of PBS, LPS (1 mg/kg) or BG (20 mg/kg) by i.p. injection. After 3 h, 1 day and 2 days, animals were terminated. **b** Microglia were FACS-isolated and gene expression levels of *Tnf*, *Il6, Il1β*, *Ccl2*, *Ccl3* and *Csf1* were detected by qPCR and normalized to *Gapdh* gene expression levels (n = 4 mice). **c** Expression levels of BG receptor gene *Dectin-1* and LPS receptor gene *Tlr4* were determined by qPCR and normalized to *Gapdh* gene expression levels (n = 4 mice). Statistical significance was determined with a one-way ANOVA followed a Bonferroni correction for multiple comparisons. *, *p* < 0.05; **, *p* < 0.01; ***, *p* < 0.001.

To investigate the effects of a systemic BG challenge on microglia *in vivo*, mice received an i.p. injection with 20 mg/kg BG dissolved in PBS. Mice injected with LPS (1 mg/kg) and PBS were included as positive and negative controls, respectively. Mice were terminated 3 h, 1 and 2 days after respective injections (Fig. 1a). Microglia were FACS-isolated from the brains, and cytokine gene expression levels were determined by qPCR. At 3 h after an LPS injection, microglia exhibited a classical inflammatory response, showing significantly increased expression of *Tnf*, *Il6, Il1β*, *Ccl2*, *Ccl3* and *Csf1* compared to PBS controls (Fig. 1b). At 2 days after LPS injection, the expression levels of these cytokine genes were back at baseline (Fig. 1b). Although we observed microglia activation in response to BG in cell culture, *in vivo* administration did not induce a significant expression of *Tnf*, *Il6, Il1β*, *Ccl2*, *Ccl3* or *Csf1* in microglia at any timepoint investigated, an observation in accordance with previous *in vitro* work [29] (Fig. 1b). However, at 3 h after BG injection, *Dectin-1* gene expression levels were significantly decreased in microglia compared to PBS controls (Fig. 1c), indicating systemic BG can activate microglia. Like BG, LPS downregulated *Dectin-1* gene expression at 3 h and 1 day after injection (Fig. 1c). *Tlr4* expression levels were not significantly altered by BG or LPS.

### BG does not induce morphological changes in cortical and hippocampal microglia

**Fig. 2.**
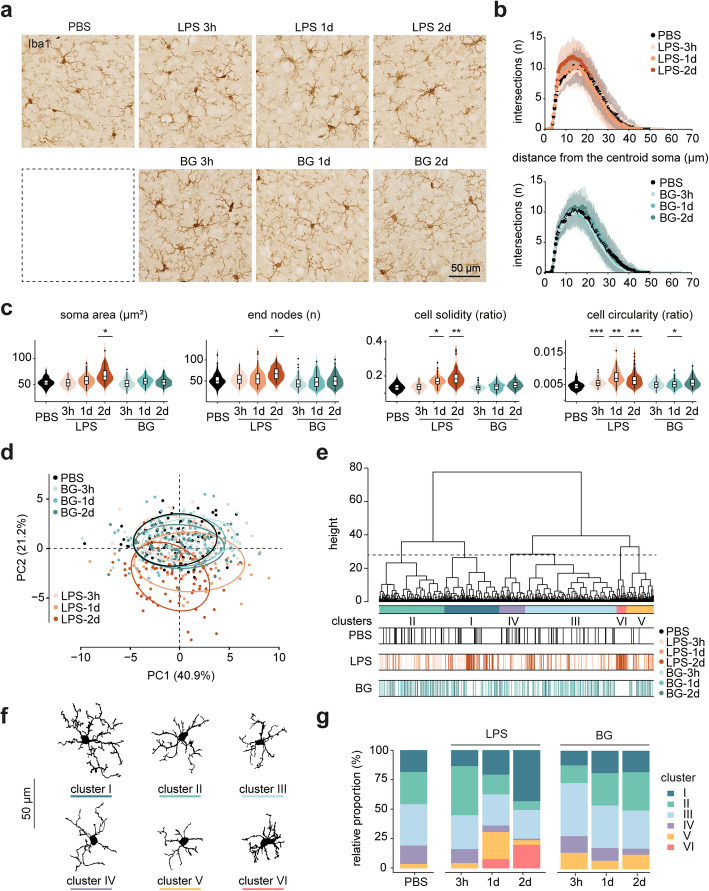
BG does not induce morphological changes in cortical microglia. **a** Representative images of Iba1-staining in the cortex across groups (scale bar: 50 μm). **b** Violin plots of representative morphometric parameters for cortical microglia across experimental groups. Violin plots represent the kernel densities for each group. Upper, middle and lower hinges of the boxplots represent the first, second (median) and third quantiles, respectively. Whiskers extend to the minimum and maximum value still within the 1.5 x the interquartile range of the first and third quantiles, respectively. Black dots represent outliers. The significance was determined by Wald chi-square test for linear mixed models. *, *p* < 0.05; **, *p* < 0.01; ***, *p* < 0.001. **c** Sholl analysis of cortical microglia at 3 h, 1 day and 2 days after LPS or BG injection. PBS-injected mice microglia served as the controls. The vertical lines represent +/- standard deviations. **d** PCA plot depicts all individual cells selected in cortex on principal component plane. The x and y axes represent the first and second principal components (PC1 and PC2), respectively. **e** Hierarchical clustering on principal components resulted in 6 cell clusters (I-VI). The dashed line indicates the cut off for 6 clusters. **f** Representative cells from each cluster are shown. **g** Cluster distribution analysis of cortical microglia at 3 h, 1 day and 2 days after LPS or BG injection. Animals with PBS injection were used as control. The number of microglia selected in the different groups: PBS: 80 cells; LPS-3h: 67 cells; LPS-1d: 63 cells; LPS-2d: 75 cells; BG-3h: 90 cells; BG-1d: 84 cells; BG-2d: 80 cells (n = 3 mice for each experimental group).

Next, microglia morphological changes in response to LPS and BG injections were investigated in the cortex by Iba1 immunostaining. At 3 h after LPS injection, no obvious morphological changes in cortical microglia were detected (Fig. 2a). At 1 and 2 days after LPS injection, pronounced morphological changes were observed, characterized by increased soma size and retracted processes compared to PBS controls (Fig. 2a). For BG, no obvious changes were observed at 3 h, 1 and 2 days after injection (Fig. 2a). To compare the degree of microglia ramifications across groups, we performed Sholl analyses. At 2 days after LPS injection, an increased number of intersections in the range of 5-20 μm distance from the soma was observed (Fig. 2b). In contrast, BG did not affect microglia ramification (Fig. 2b). Summarizing, these results indicate that an i.p. LPS challenge significantly affected cortical microglia morphology, and that an i.p. BG injection had limited effects. To quantify morphological changes in microglia, we used a self-developed pipeline to perform morphometric analysis on microglia, randomly selected in the cortex across different groups. Additional file 4 contains detailed morphometric information of all selected cells and statistical comparisons between different experimental groups in cortical microglia for all features measured. At 3 h after LPS injection, only cell circularity values were significantly increased compared to PBS controls (Fig. 2c and Additional file 4). At 2 days after LPS injection, more morphological features, such as soma area, the number of end nodes and cell solidity were significantly changed compared to PBS controls (Fig. 2c and Additional file 4). In contrast, the effect of BG on morphological features of microglia was very limited (Fig. 2c and Additional file 4).

Next, we performed principal component analysis (PCA) to reduce the dimensionality of the cortex microglia morphometric dataset. Extensive overlap between microglia from BG-treated groups and PBS-treated group was observed (Fig. 2d), indicating similar microglia morphologies. In contrast, some microglia in the LPS-1d and LPS-2d groups segregated in the PCA plot (Fig. 2d), indicating morphological differences. To identify microglia subsets with similar morphologies, we performed a hierarchical clustering on the main contributing principal components, as described in methods section (Additional file 2), resulting in 6 microglia clusters (I-VI) with distinct morphological properties (Fig. 2e). The representative cell silhouettes for each cluster are depicted in Fig. 2f. Cells in cluster I and II were characterized by relatively long branch lengths and small soma sizes (Fig. 2f). In contrast, cells in cluster VI had a relatively large soma size and short branch length (Fig. 2f) and were exclusively derived LPS-treated groups (Fig. 2e). Next, we analyzed the relative distribution of these distinct microglia clusters over the respective treatment groups. In PBS-injected mice, microglia clusters I-V were present, indicating microglia were morphologically heterogeneous even under homeostatic conditions (Fig. 2g). At 3 h after LPS injection, microglia cluster distributions were only mildly affected compared to control conditions (Fig. 2g). However, at 1 and 2 days after LPS injection, the relative proportion of cluster I microglia increased, cluster II microglia were considerably reduced and cluster VI microglia appeared (Fig. 2g). In contrast, at 3h, 1 and 2 days after BG injection, the distribution of the different microglia clusters was only slightly different compared to control conditions (Fig. 2d,g). These results indicated that LPS induced a subset of microglia with a reactive morphology in cortex, whereas this subset of microglia was not detected in response to BG.

Given the regional heterogeneity of microglia [37, 38], microglia cells in the hippocampus were also randomly selected and analyzed. Additional file 4 provides detailed morphometric information of all selected cells and statistical comparisons between different experimental groups in the hippocampus for all features measured. Similarly, BG induced only mild morphological changes in hippocampal microglia, while an LPS challenge, like was observed in the cortex, resulted in a subset of microglia with a morphologically reactive profile (Additional file 4 and 5). In summary, these results indicate that where a systemic LPS challenge resulted in extensive morphological changes in microglia, a BG challenge did not result in altered cortical and hippocampal microglia morphology.

### Systemic administration of BG induces immune training in microglia

**Fig. 3.**
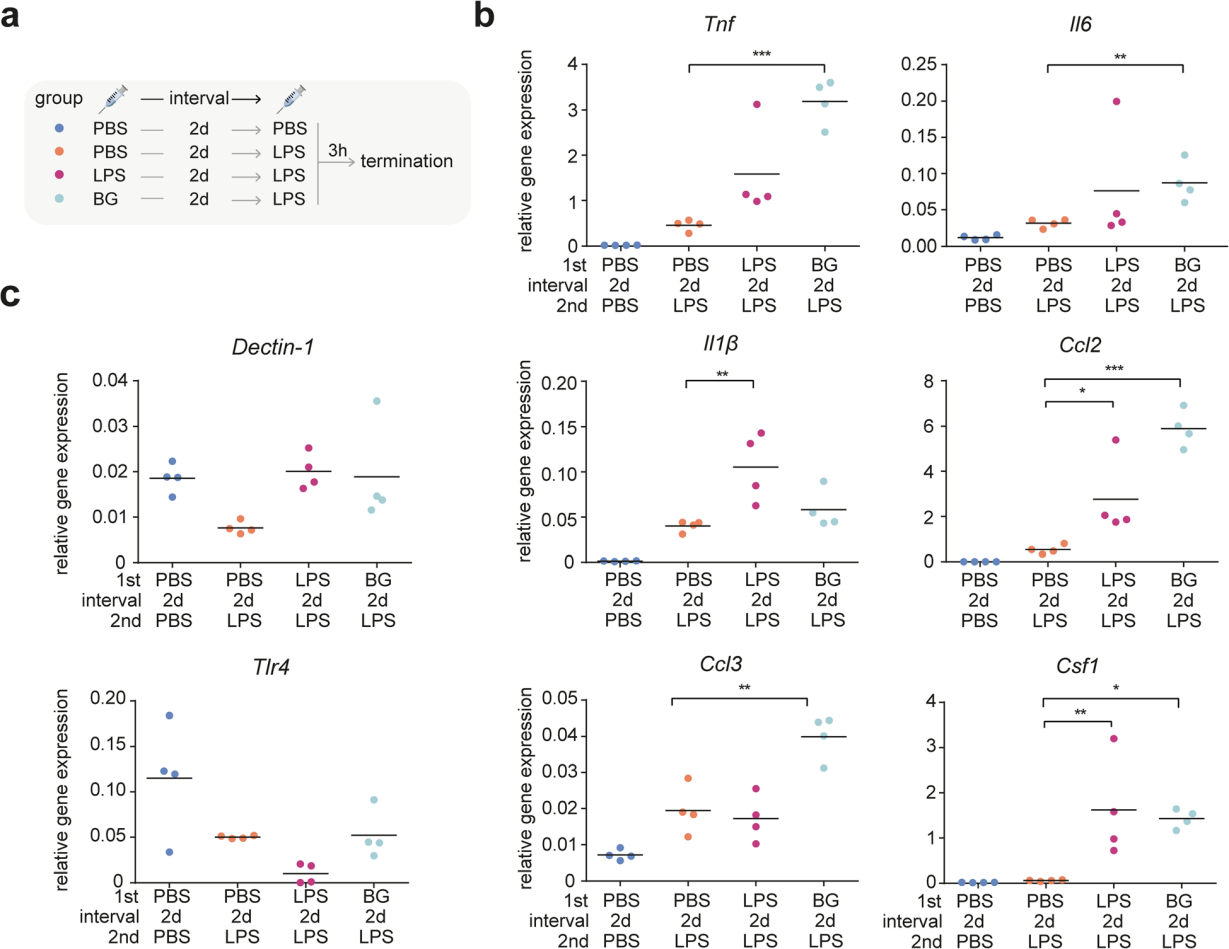
BG induces immune training in microglia 2 days after injection. **a** Diagram of preconditioning experimental design with 2-day interval. Mice were first challenged with PBS, LPS (1 mg/kg) or BG (20 mg/kg) by i.p. injection on day 0. On day 2, all the mice received a second stimulation of PBS or LPS (1 mg/kg). Animals were terminated 3 h after the second injection. **b** Microglia were isolated and gene expression levels of *Tnf, Il1β, Ccl2, Ccl3, Il6* and *Csf1* were determined using qPCR and normalized to *Gapdh* gene expression levels (n = 4 mice). **c** Gene expression levels of *Dectin-1* and *Tlr4* were determined by qPCR and normalized to *Gapdh* gene expression levels (n = 4 mice). The significance was calculated by one-way ANOVA followed a Bonferroni correction for multiple comparisons. *, *p* < 0.05; **, *p* < 0.01; ***, *p* < 0.001.

To determine if BG induced immune memory in microglia *in vivo*, we first challenged mice with BG or LPS, and after 2 days, we challenged the mice with LPS (Fig. 3a). For controls, mice were injected with PBS and after 2 days challenged with PBS of LPS. BG preconditioning resulted in an increased transcriptional response to LPS (Fig. 3b), indicating BG induced immune training of microglia. Interestingly, with a 2-day interval between the first and second challenge, LPS preconditioning also induced immune training in microglia (Fig. 3b, LPS-2d-LPS vs. PBS-2d-LPS group). In line with these findings, it was shown that LPS preconditioning induced immune training in microglia 1 day after the first challenge [28]. Nevertheless, LPS and BG trained microglia in different ways: In terms of *Tnf*, *Il-6* and *Ccl3* expression, only BG preconditioning significantly trained microglia (Fig. 3b); but for *Il-1β* expression, only LPS could significantly train microglia (Fig. 3b); for *Ccl2* and *Csf1* expression, both LPS and BG could significantly train microglia (Fig. 3b). Since microglia cytokines gene expression levels were back at baseline, 2 days after the first injection (Fig. 1b,c), the observed enhanced responsiveness to a second LPS challenge was not due to a cumulative increase in gene expression levels as a result of the first stimulation. Taken together, LPS-and BG-induced immune training of microglia was detected *in vivo*, 2 days after the preconditioning stimulus.

Microglia gene expression levels of the BG and LPS receptors, Dectin-1 and Tlr4*,* respectively, were determined. Acute LPS stimulation resulted in near-significant downregulation of *Tlr4* and *Dectin-1* expression in microglia (Fig. 3c, PBS-2d-LPS vs. PBS-2d-PBS group). However, after LPS and BG preconditioning, a second LPS injection did not result in reduced *Dectin-1* expression (Fig. 3c, LPS-2d-LPS and BG-2d-LPS vs. PBS-2d-LPS group), suggesting preconditioning dampened the transcriptional response of *Dectin-1* to an LPS challenge. For *Tlr4* expression*,* BG preconditioning did not alter its expression in response an LPS challenge, where LPS preconditioning seemed to further reduce *Tlr4* expression in response to LPS (Fig. 3c).

Next, we analyzed the persistence of BG induced immune memory. We first challenged mice with 20 mg/kg BG, and after 2, 7 or 14 days, we challenged the mice with 1 mg/kg LPS (Additional file 6a). For the control groups, mice were first challenged with PBS, and at 7 days after the first injection, mice were re-challenged with PBS or 1 mg/kg LPS (Additional file 6a). We also included an LPS preconditioning group, as we previously reported that LPS preconditioning resulted in immune tolerance in microglia after 7 days [27]. Three hours after the second injection, mice were terminated (Additional file 6a), microglia were isolated and qPCRs were performed. In accordance with our previous findings [27], LPS preconditioning induced immune tolerance in microglia 7 days later, reflected by reduced expression of *Tnf*, *Il6, Il1β*, *Ccl2*, *Ccl3* and *Csf1* to a subsequent LPS stimulation (Additional file 6b, LPS-7d-LPS vs. PBS-7d-LPS group). Preconditioning by i.p. BG injection resulted in enhanced expression of *Tnf*, *Il6, Il-1β* and *Csf1* 2 days later (Additional file 6b, BG-2d-LPS vs. PBS-7d-LPS group). However, at 7 or 14 days after the first BG challenge, this BG immune training phenotype was no longer detected (Additional file 6b).

### LPS- and BG-preconditioned mice microglia display an enhanced reactive morphology in response to a second LPS injection

**Fig. 4.**
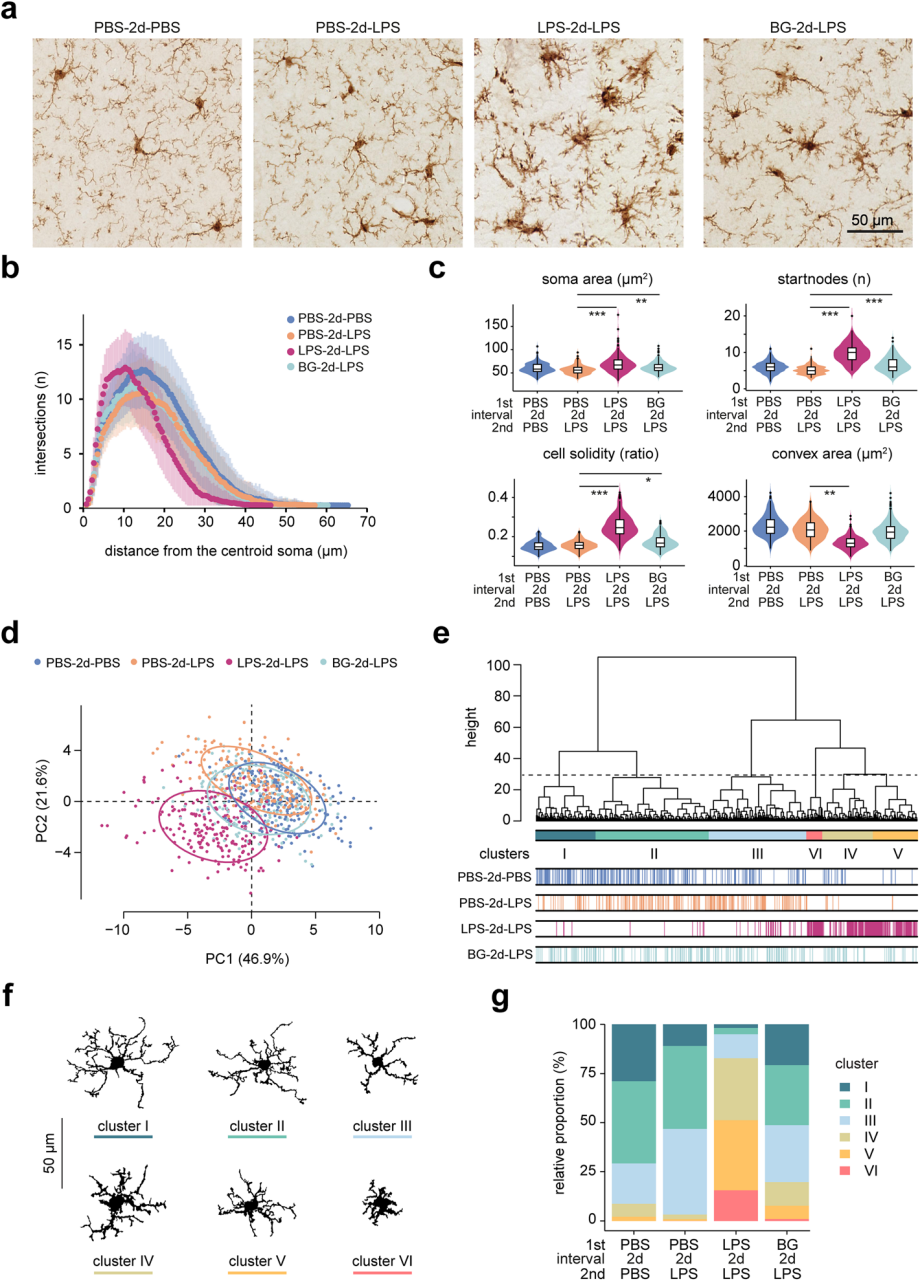
LPS- and BG-preconditioned mice microglia display an exaggerated reactive morphotype in response to a second LPS injection in cortex. **a** Representative images of Iba1-staining in the cortex across different groups (scale bar: 50 μm). **b** Violin plots of representative morphometric parameters for cortical microglia across experimental groups. The significance was determined by Wald chi-square test for linear mixed models. *, *p* < 0.05; **, *p* < 0.01; ***, *p* < 0.001. **c** Sholl analysis of cortical microglia across groups. The vertical lines represent +/- standard deviations. **d** PCA plot depicts all individual cells selected in cortex on principal component plane. The x and y axes represent the first and second principal components (PC1 and PC2), respectively. **e** Hierarchical clustering on principal components resulted in 6 cell clusters (I-VI). The dashed line indicates the cut off for 6 clusters. **f** Representative cells from each cluster are shown. **g** Cluster distribution analysis of LPS- and BG-preconditioned mice microglia 3 h after second LPS challenge in cortex. Microglia from naïve mice with two PBS injections (PBS-2d-PBS group) and mice that only received one LPS challenge for 3 h (PBS-2d-LPS group) were included as controls. The number of microglia selected in the different groups: PBS-2d-PBS: 228 cells; PBS-2d-LPS: 210 cells; LPS-2d-LPS: 220 cells; BG-2d-LPS: 208 cells (n= 4 mice for each experimental group).

Next, we performed morphometric analysis of Iba1-stained microglia in the cortex of LPS- and BG-preconditioned mice that received a second LPS injection. In the cortex, at 3h after a second LPS injection, we observed prominent morphological changes in microglia in LPS preconditioned mice (LPS-2d-LPS group) compared to controls (PBS-2d-PBS group) or mice that only received the second LPS challenge (PBS-2d-LPS group) (Fig. 4a). In BG-preconditioned mice, some microglia also exhibited pronounced morphological changes in response to a second LPS injection (Fig. 4a), but less robust compared to LPS-preconditioned microglia. Sholl analysis showed that LPS-2d-LPS group microglia had more intersections at ~5 μm to ~15 μm distance from the soma and a shorter maximum branch radius compared to PBS-2d-LPS group (Fig. 4b). For the microglia in the BG-2d-LPS group, the branching profile was quite similar to the PBS-2d-LPS group (Fig. 4b). Soma area, the number of start nodes, cell solidity and convex area were all significantly altered in LPS-2d-LPS microglia (Fig. 4c). Detailed morphometric information of all selected cells and statistical comparisons between different experimental groups in cortex for all features measured are provided in Additional file 7. These results indicate that both LPS- and BG-preconditioning resulted in increased morphological changes in microglia to a second LPS challenge, and these changes were most pronounced in LPS-preconditioned mice.

PCA was performed on the cortical microglia morphometric dataset. An extensive overlap was observed between microglia from PBS-2d-LPS group and PBS-2d-PBS group (Fig. 4d), indicating similar morphologies. In contrast, some BG- but especially LPS-preconditioned microglia were more segregated from PBS-2d-PBS microglia (Fig. 4d). Next, we performed hierarchical clustering on the main contributing principal components, resulting in 6 microglia clusters (I-VI) with distinct morphological properties (Fig. 4e). Cell silhouettes representative for each cluster are depicted (Fig. 4f). Cells in cluster I and II had a relatively long branch length and small soma size; cells in cluster IV, V and VI showed a typical activated morphology with a relatively large soma size and short branch length (Fig. 4f). Next, we analyzed the relative distribution of these distinct microglia clusters over the respective treatment groups. Similar to our previous finding (Fig. 2g), 3 h after an acute LPS stimulation, microglia cluster distribution was only mildly affected compared to control conditions (Fig. 4g, PBS-2d-LPS vs. PBS-2d-PBS group). However, in LPS preconditioned mice, the percentages of clusters IV, V and VI microglia considerably increased (Fig. 4g). In the BG-2d-LPS group, an increase in cluster IV, V and VI microglia was observed compared to control (PBS-2d-PBS) and acute LPS stimulated mice (PBS-2d-LPS) but much less than was observed in the LPS-2d-LPS group (Fig. 4g). These results indicate that compared to PBS-preconditioned mice microglia, a subset of microglia acquired a reactive morphology after a second LPS injection in LPS- and BG-preconditioned mice.

Similar results were also observed in the hippocampus region (Additional file 8), demonstrating that these morphological changes were not restricted to the cortex. Additional file 7 provides detailed morphometric information of all selected cells and statistical comparisons between different experimental groups in the hippocampus. In summary, LPS- and BG-preconditioned mice microglia display an increased reactive morphology in response to a second LPS injection in cortex and hippocampus.

### Systemic administration of BG and LPS induces immune tolerance in the periphery

**Fig. 5.**
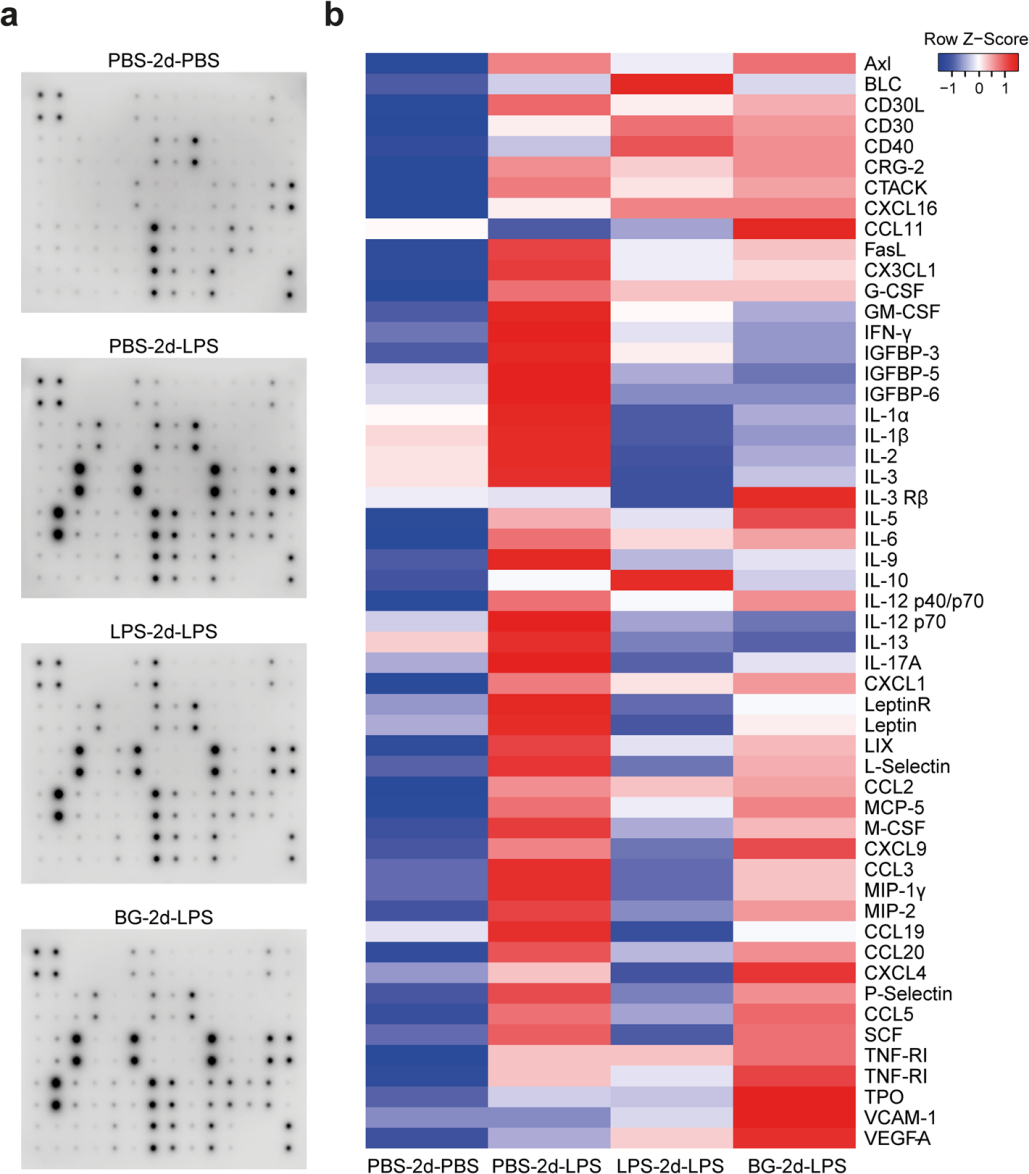
Peripheral immune tolerance in LPS- and BG-preconditioned mice. Mice were first challenged with PBS, LPS (1 mg/kg) or BG (20 mg/kg) by i.p. injection. Two days later, the mice received a second stimulation with PBS or LPS (1 mg/kg). Animals were terminated 3 h after the second injection. Sera of mice in the same experimental group were pooled (n = 4 for each group). Cytokine levels were measured with a cytokine array (**a**). Densitometric analysis of the immunoreactivity of each cytokine or chemokine was performed. After filtering, a heatmap was generated based on optical density levels of cytokines and chemokines (**b**).

In this study, microglia innate immune memory was induced by systemic administration of LPS or BG. However, previous studies have shown that systemic challenges can also alter the epigenetic signature of peripheral immune cells and their response to subsequent stimuli [9–11]. To determine whether the peripheral response to LPS is affected by preconditioning, serum cytokine concentrations of BG- and LPS-preconditioned mice were determined 3 h after the second LPS challenge by means of a cytokine array (Fig. 5a). Similar to previous findings [28], LPS induced peripheral immune tolerance 2 days after the first LPS injection, as evidenced by decreased serum concentrations of IL-1β, IFN-γ and IL-12 p70 when compared to cytokine serum concentrations of mice exposed to a single LPS injection (Fig. 5b, LPS-2d-LPS vs. PBS-2d-LPS group). Interestingly, in the BG-2d-LPS group, serum concentrations of pro-inflammatory cytokines such as IL-1β, IFN-γ, IL-12 p70 and IL-6 were reduced when compared to PBS-2d-LPS mice (Fig. 5b), suggesting BG also induced peripheral immune tolerance. These results show that with an interval of 2 days between the first and second challenge, LPS and BG preconditioning both induced immune tolerance in the periphery, suggesting that the immune training observed in microglia is most likely driven by microglia-intrinsic changes, possibly by epigenetic alterations as reported previously [28]. However, a contribution of peripheral immune cells cannot be ruled out as some peripheral cytokines or chemokines like IL-5, CXCL4 and CXCL9 also showed an enhanced response to LPS, 2 days after a BG challenge (Fig. 5b).

## Discussion

Microglia, the resident macrophages of the CNS, play an important role in brain development and homeostasis. Infections or any other disturbances to the homeostasis of the brain induce immediate activation of microglia, which is accompanied by the secretion of cytokines, production of ROS, altered phagocytic activities and morphological changes [39]. The discovery of innate immune memory in microglia has revealed an important and previously unrecognized CNS immune property, showing that, besides the local environment, previous inflammatory experiences can affect the microglial response to a sequential challenge [26]. LPS and BG are two commonly used immune modulators in monocytes and macrophages [3, 5, 19]. Several studies have confirmed that LPS preconditioning can induce immune training and tolerance in microglia *in vivo* and *in vitro* [27, 28, 40]. Compared to LPS, the effects of BG on microglia innate immune memory are poorly understood, especially *in vivo*. Notably, BG induced immune training in microglia, as mice preconditioned with BG displayed an enhanced responsiveness to a subsequent LPS exposure, as evidenced by the increased cytokine gene expression and morphological changes in microglia.

In our previous study, we reported that systemically administered LPS induced microglia immune tolerance 7 days later *in vivo* [27]*,* which is also confirmed in this study. In addition, we previously showed that LPS-induced immune tolerance in microglia could last at least 32 weeks after the first challenge [27]. However, with the same dose of LPS (1 mg/kg), we observed immune training instead of immune tolerance 2 days after the first injection. With the dose of BG used in this study, we only observed immune training in microglia 2 days after preconditioning, and this training phenotype was no longer detectable at 7 and 14 days after the first BG challenge. These results indicate that the time interval between the first and second challenge is crucial to unravel microglia innate immune memory *in vivo*. In addition, different LPS treatment paradigms were reported to induce different microglia innate immune memories in microglia [28]. A single i.p. LPS injection (0.5 mg/kg) caused an immune training response 24 h after, while 4 daily, consecutive LPS injections (with the same dose) induced immune tolerance in microglia [28]. More systemic investigations are needed to delineate how different intervals between challenges, the pathogen type, its dose and the number of exposures affect the microglia innate memory response.

Wendeln et al. showed that 6 months after a desensitizing stimulation, tolerized microglia showed significant loss of H3K4me1 on enhancers of genes related to PI3K-Akt signaling pathway [28], suggesting PI3K-Akt signaling is suppressed during microglia immune tolerance. In a recent *in vitro* study, Lajqi et al. showed that, in response to a second LPS challenge, TLR4, MyD88 and phospho-p65 were more responsive in trained microglia induced by an ultralow LPS dose (1 fg/mL) of LPS compared to naïve microglia, and that immune training is mediated by the PI3K-Akt signalling pathway [40]. Based on these results, it is possible that the first BG injection also induced immune training in microglia via the PI3K-Akt and NF-κB pathways.

Our cytokine array results show that with an interval of 2 days between the first and second challenge, LPS and BG preconditioning both induced peripheral immune tolerance. In a previous study [24], BG preconditioning was shown to induce immune training in the periphery. This difference might have several causes. First, the time interval between two challenges was different (2 days in this study, 4 days in the previous study). Second, in the previous study, BG from *C. albicans* was used*,* while we used BG from *S. cerevisiae*. And it is known that the immune stimulatory effects of BGs are linked to their structural complexity which is highly related to the BG source [18, 21]. Third, it has been shown *in vitro* that the degree and type of innate immune response in monocytes also depends on the concentration of the ligand the cells are exposed to [41]. In our study, we used a slightly lower dose of BG (20 mg/kg in this study, 1 mg/animal in the previous study). Recently, Wendeln et al. showed that one individual cytokine, TNF-α, applied peripherally also could induce immune memory in the brain [28]. Peripheral cytokine profiles need to be further characterized by multiplex cytokine assays after a first challenge. Then, the potential cytokine or chemokine induced by the first challenge could be applied individually or blocked by neutralizing antibodies in the periphery, to study the influence of individual cytokines or chemokines on microglia innate immune memory. And conversely, it would also be a useful experiment to employ mice lacking receptor such as TLR4 or for a specific cytokine conditionally only in microglia. These experiments would improve our knowledge of the crosstalk between the periphery and the brain in microglia innate immune memory. It is difficult to separate microglia immune training from the peripheral response in vivo and to identify the responsible peripheral cell types. It is known that a peripheral LPS challenge can epigenetically alter microglia, and change their response to a second challenge. However, also the peripheral response to a second challenge can be altered by a preconditioning stimulus. As a consequence, the response of microglia to a challenge in preconditioned mice is probably the result of an altered microglia response (due to intrinsic i.e. epigenetic) changes in combination with an altered peripheral response.

As long-lived tissue macrophages in the CNS, microglia innate immune memory induced by a previous stimulus might have long term functional impacts. We previously showed that LPS-induced immune tolerance in microglia could at least last for 32 weeks [27]. Wendeln et al. showed that microglia are transcriptionally and epigenetically altered even 6 months after LPS challenges [28]. LPS-induced immune training exacerbated pathology and LPS-induced immune tolerance alleviated cerebral β-amyloidosis [28]. This beneficial effect of immune tolerance was also observed in a stroke mouse model [28]. Recently, in primary microglia, chronic exposure (twice a 24 h exposure with an interval of 3-5 days) to Aβ induced immune tolerance and metabolic defects. This defect was characterized by reduced glycolysis and oxidative phosphorylation compared to microglia exposed only to Aβ once [42]. In addition, metabolic boosting with IFN-γ could restore immunological function of microglia [42]. Similarly, in monocytes, β-glucan partially reversed LPS-induced immune tolerance through epigenetic regulation [5]. In this study, we showed that β-glucan could induce immune training in microglia. It would be interesting to investigate whether β-glucan or other immune modifiers could reverse the immune state of microglia in disease conditions. Thus, modulating innate immune memory in microglia is a promising target to develop treatment for microglia-associated diseases.

## Conclusion

Our results show that BG activated microglia without inducing significant cytokine expression. BG- and LPS-preconditioning both induced immune training in microglia two days after the first challenge. However, with an interval of 7 days between the first and second challenge, LPS-preconditioning induced immune tolerance in microglia where BG-induced immune training was no longer detected.

## Supplementary Information

**Additional file 1.** Information on qPCR primers.

**Additional file 2.** Categorization of microglia with similar shape and size by means of hierarchical clustering on principal components (PCs). Here we used the acute design cortex dataset as an example. To reduce the dimensionality of the dataset, a PCA was performed. a) Table depicts the eigenvalues, the variance retained and the cumulative variance for the first 10 PCs. PCs with an eigenvalue > 1 (here, PC1-PC5) were retained for hierarchical clustering. b) Hierarchical clustering on the first 5 PCs resulted in 6 distinct morphological clusters (I-VI). c) Heatmap represents the z-scores for all morphometric features for microglia clusters I-VI.

**Additional file 3 **BG and LPS activate NF-κB signaling in a BV-2 cell line and induce cytokine gene expression in primary microglia. a) A BV-2 cell line carrying a NF-κB -luciferase reporter gene was stimulated by LPS (100 ng/mL) or BG (10 μg/ml) for the times indicated (ranging from 15 min to 24 h). Luciferase activity was determined and normalized to PBS treated cells. b) BV-2 cells carrying a NF-κB-luciferase reporter gene were stimulated for 4 h with different concentrations of LPS or BG. **c**) Primary microglia were stimulated by LPS (100 ng/mL) or BG (10 μg/ml). After 3 h, cells were washed by PBS and RNA was isolated. Expression levels of *Tnf*, *Il6, Il1β*, *Ccl3* and *Csf1* were determined by qPCR and normalized to *Hprt1* gene expression levels. Statistical significance was determined with a one-way ANOVA followed a Bonferroni correction for multiple comparisons. *, *p* < 0.05; **, *p* < 0.01; ***, *p* < 0.001.

**Additional file 4.** Raw morphometric information for all cells analyzed in acute design, cortex and hippocampus regions. Statistical comparisons between experimental groups are also provided.

**Additional file5 **BG does not induce morphological changes in hippocampal microglia. a) Representative images of Iba1-staining in the hippocampus across groups (scale bar: 50 μm). b) Violin plots of representative morphometric parameters for cortical microglia across experimental groups. The significance was determined by Wald chi-square test for linear mixed models. *, *p* < 0.05; **, *p* < 0.01; ***, *p* < 0.001. c) Sholl analysis of hippocampal microglia at 3 h, 1 day and 2 days after LPS or BG injection. PBS-injected mice microglia served as the controls. The vertical lines represent +/- standard deviations. d) PCA plot depicts all individual cells selected in hippocampus on principal component plane. The x and y axes represent the first and second principal components (PC1 and PC2), respectively. e) Hierarchical clustering on principal components resulted in 6 cell clusters (I-VI). The dashed line indicates the cut off for 6 clusters. f) Representative cells from each cluster are shown. g) Cluster distribution analysis of hippocampal microglia at 3 h, 1 day and 2 days after LPS or BG injection. Animals with PBS injection were used as control. The number of microglia selected in the different groups: PBS: 42 cells; LPS-3h: 49 cells; LPS-1d: 51 cells; LPS-2d: 48 cells; BG-3h: 39 cells; BG-1d: 49 cells; BG-2d: 51 cells (n = 3 mice for each experimental group).

**Additional file 6 **BG induces immune training in microglia *in vivo* 2 days after the first injection. a) Diagram of preconditioning experimental design. Mice received first challenge of PBS, LPS (1 mg/kg) or BG (20 mg/kg) by i.p. injection. After 2, 7 or 14 days, the same mice received second challenge with PBS or LPS (1 mg/kg). Animals were terminated 3 h after the second injection. b) Microglia were isolated and *Tnf, Il6*, *Il-1β*, *Ccl2*, *Ccl3* and *Csf1* gene expression levels were detected by qPCR and normalized to *Hprt1* gene expression levels (*n* = 3 mice).

**Additional file 7.** Raw morphometric information for all cells analyzed in preconditioning design, cortex and hippocampus regions. Statistical comparisons between experimental groups are also provided.

**Additional file 8.** LPS- and BG-preconditioned mice microglia display an exaggerated reactive morphotype in response to a second LPS injection in hippocampus. **a**) Representative images of Iba1-staining in the hippocampus across different groups (scale bar: 50 μm). **b**) Violin plots of representative morphometric parameters for hippocampal microglia across experimental groups. The significance was determined by Wald chi-square test for linear mixed models. *, *p* < 0.05; **, *p* < 0.01; ***, *p* < 0.001. **c**) Sholl analysis of hippocampal microglia across groups. The vertical lines represent +/- standard deviations. **d**) PCA plot depicts all individual cells selected in hippocampus on principal component plane. The x and y axes represent the first and second principal components (PC1 and PC2), respectively. **e**) Hierarchical clustering on principal components resulted in 6 cell clusters (I-VI). The dashed line indicates the cut off for 6 clusters. **f**) Representative cells from each cluster are shown. **g**) Cluster distribution analysis of LPS- and BG-preconditioned mice microglia 3 h after second LPS challenge in hippocampus. Microglia from naïve mice with two PBS injections (PBS-2d-PBS group) and mice that only received one LPS challenge for 3 h (PBS-2d-LPS group) were included as controls. The number of microglia selected in the different groups: PBS-2d-PBS: 139 cells; PBS-2d-LPS: 138 cells; LPS-2d-LPS: 144 cells; BG-2d-LPS: 137 cells (n= 4 mice for each experimental group).

## Data Availability

The datasets supporting the conclusion of this article are included within the article (and its additional files). The datasets generated and/or analyzed during the current study are stored in a public repository and are available from the corresponding authors on reasonable request.

## Abbreviations

AR: Antigen retrieval
BBB: Blood brain barrier
BG: β-glucan
CNS: Central nervous system
DAB: 3,3’-Diaminobenzidine
FACS: Fluorescence-activated cell sorting
HIF1α: Hypoxia-inducible factor-1α
Iba1: Ionized calcium-binding adapter molecule 1
LPS: Lipopolysaccharide
NDS: Normal donkey serum
NF-κB: Nuclear factor-κB
PAMP: Pathogen-associated molecular pattern
PBS: Phosphate buffered saline
PCA: Principal component analysis
PFA: Paraformaldehyde
ROS: Radical oxygen species
RT: Room temperature
SAE: Sepsis-associated encephalopathy
TLR: Toll-like receptor
