# Supplementary material for: Systemic administration of β-glucan induces immune training in microglia
Source: J Neuroinflammation. 2021 Feb 22;18:57. doi: 10.1186/s12974-021-02103-4 (PMC7901224; doi:10.1186/s12974-021-02103-4)
Supplement: Supplementary file 1 — Additional file 1. Information on qPCR primers. [file 12974_2021_2103_MOESM1_ESM.docx]

**Additional Figure: Table S1. qPCR primer sequences**

| **Gene name** | **Forward primer 5’-3’** | **Reverse primer 5’-3’** |
| --- | --- | --- |
| *Gapdh* | CATCAAGAAGGTGGTGAAGC | ACCACCCTGTTGCTGTAG |
| *Hprt1* | ATACAGGCCAGACTTTGTTGGA | TGCGCTCATCTTAGGCTTTGTA |
| *Tnf* | TCTTCTGTCTACTGAACTTCGG | AAGATGATCTGAGTGTGAGGG |
| *Il1b* | CCCAAAAGATGAAGGGCTGC | TGATACTGCCTGCCTGAAGC |
| *Ccl2* | TCAGCCAGATGCAGTTAACG | CTGGTGATCCTCTTGTAGCTC |
| *Ccl3* | CACGCCAATTCATCGTTGAC | CTGCCGGTTTCTCTTAGTCAG |
| *Il6* | GAGGATACCACTCCCAACAGACC | AAGTGCATCATCGTTGTTCATACA |
| *Csf1* | CAAGATCTGGACAAAGAGGCCA | CCGGTGGATGCAGTTTTTAGAAG |
| *Dectin-1* | CCCAACTCGTTTCAAGTCAG | AGACCTCTGATCCATGAATCC |
| *Tlr4* | GAATCCCTGCATAGAGGTAGTTCC | TGATCCATGCATTGGTAGGTAATATTA |
