# Supplementary figures and images for: Systemic administration of β-glucan induces immune training in microglia
Source: J Neuroinflammation. 2021 Feb 22;18:57. doi: 10.1186/s12974-021-02103-4 (PMC7901224; doi:10.1186/s12974-021-02103-4)

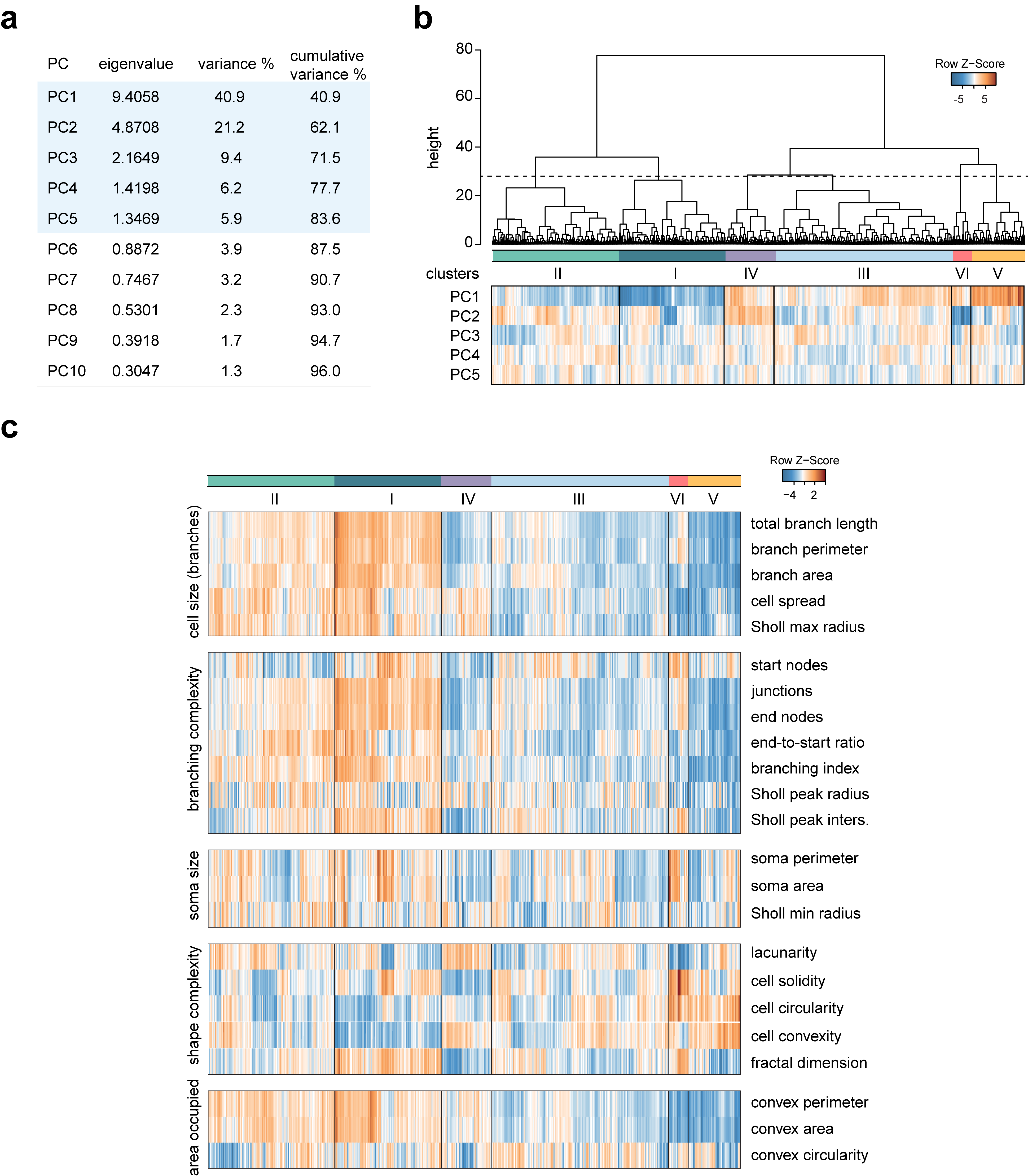

Supplement: Supplementary file 2 — Additional file 2. Categorization of microglia with similar shape and size by means of hierarchical clustering on principal components (PCs). Here we used the acute design cortex dataset as an example. To reduce the dimensionality of the dataset, a PCA was performed. a) Table depicts the eigenvalues, the variance retained and the cumulative variance for the first 10 PCs. PCs with an eigenvalue > 1 (here, PC1-PC5) were retained for hierarchical clustering. b) Hierarchical clustering on the first 5 PCs resulted in 6 distinct morphological clusters (I-VI). c) Heatmap represents the z-scores for all morphometric features for microglia clusters I-VI. [file 12974_2021_2103_MOESM2_ESM.tif]

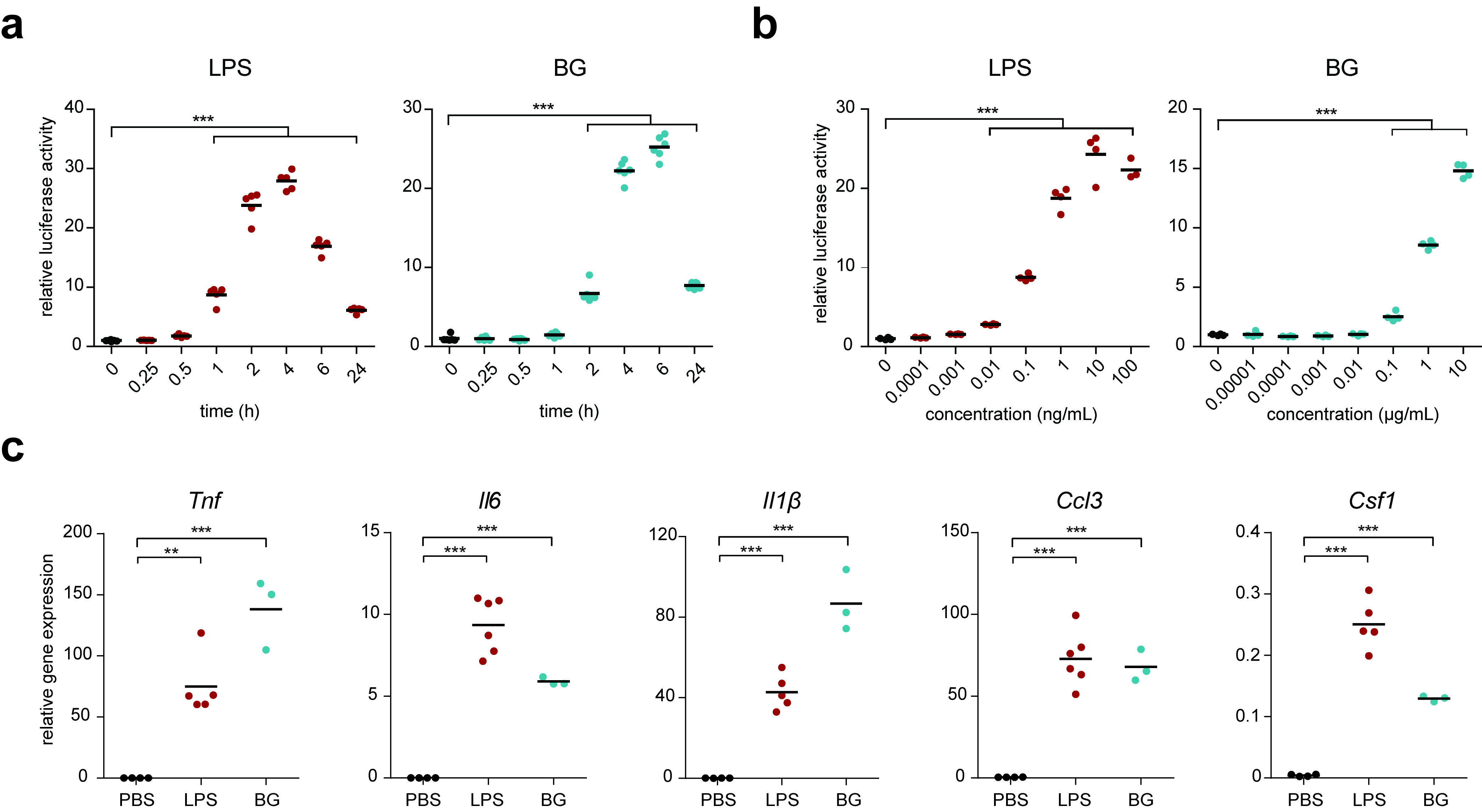

Supplement: Supplementary file 3 — Additional file 3 BG and LPS activate NF-κB signaling in a BV-2 cell line and induce cytokine gene expression in primary microglia. a) A BV-2 cell line carrying a NF-κB -luciferase reporter gene was stimulated by LPS (100 ng/mL) or BG (10 μg/ml) for the times indicated (ranging from 15 min to 24 h). Luciferase activity was determined and normalized to PBS treated cells. b) BV-2 cells carrying a NF-κB-luciferase reporter gene were stimulated for 4 h with different concentrations of LPS or BG. c) Primary microglia were stimulated by LPS (100 ng/mL) or BG (10 μg/ml). After 3 h, cells were washed by PBS and RNA was isolated. Expression levels of Tnf, Il6, Il1β, Ccl3 and Csf1 were determined by qPCR and normalized to Hprt1 gene expression levels. Statistical significance was determined with a one-way ANOVA followed a Bonferroni correction for multiple comparisons. *, p < 0.05; **, p < 0.01; ***, p < 0.001. [file 12974_2021_2103_MOESM3_ESM.tif]

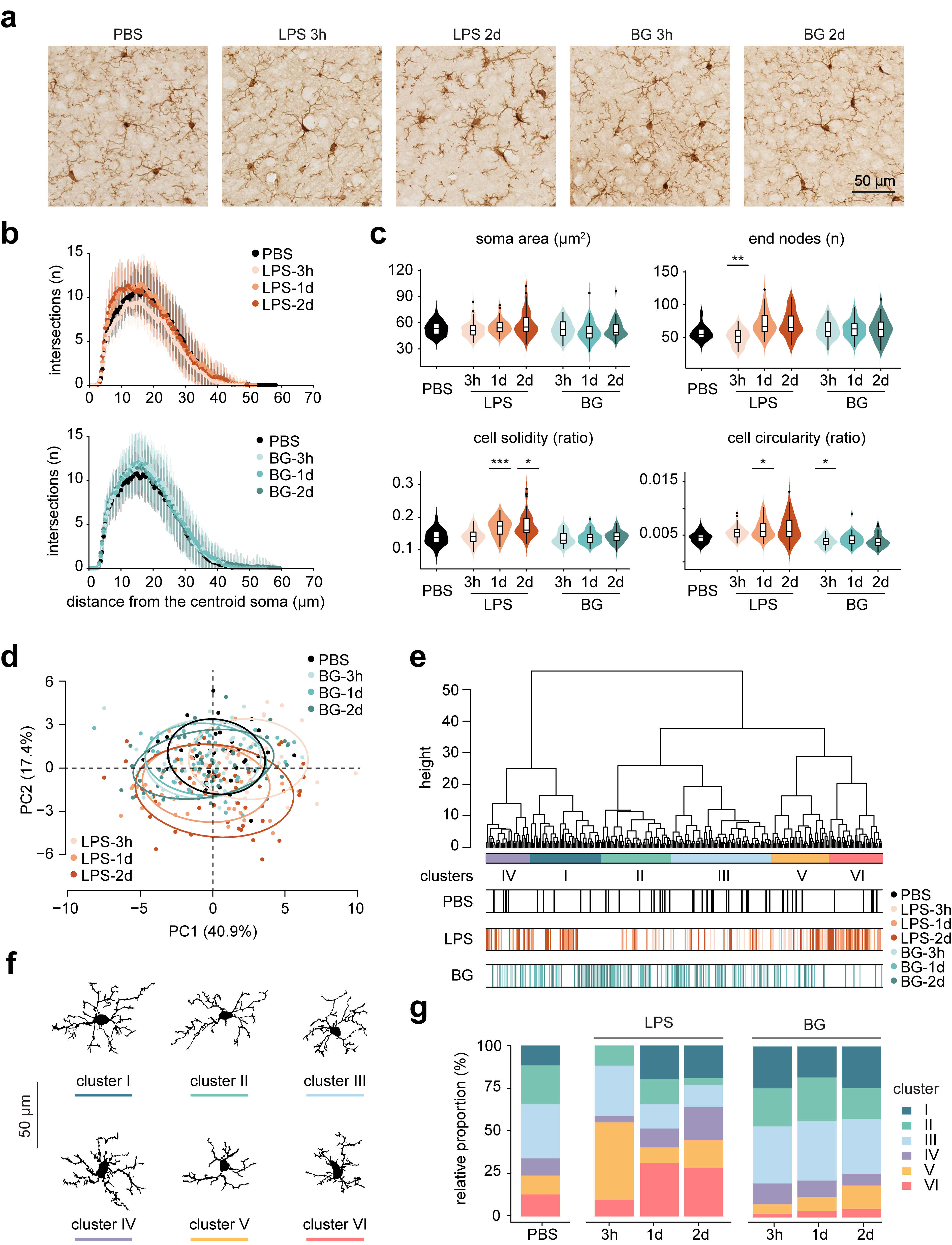

Supplement: Supplementary file 5 — Additional file5 BG does not induce morphological changes in hippocampal microglia. a) Representative images of Iba1-staining in the hippocampus across groups (scale bar: 50 μm). b) Violin plots of representative morphometric parameters for cortical microglia across experimental groups. The significance was determined by Wald chi-square test for linear mixed models. *, p < 0.05; **, p < 0.01; ***, p < 0.001. c) Sholl analysis of hippocampal microglia at 3 h, 1 day and 2 days after LPS or BG injection. PBS-injected mice microglia served as the controls. The vertical lines represent +/- standard deviations. d) PCA plot depicts all individual cells selected in hippocampus on principal component plane. The x and y axes represent the first and second principal components (PC1 and PC2), respectively. e) Hierarchical clustering on principal components resulted in 6 cell clusters (I-VI). The dashed line indicates the cut off for 6 clusters. f) Representative cells from each cluster are shown. g) Cluster distribution analysis of hippocampal microglia at 3 h, 1 day and 2 days after LPS or BG injection. Animals with PBS injection were used as control. The number of microglia selected in the different groups: PBS: 42 cells; LPS-3h: 49 cells; LPS-1d: 51 cells; LPS-2d: 48 cells; BG-3h: 39 cells; BG-1d: 49 cells; BG-2d: 51 cells (n = 3 mice for each experimental group). [file 12974_2021_2103_MOESM5_ESM.tif]

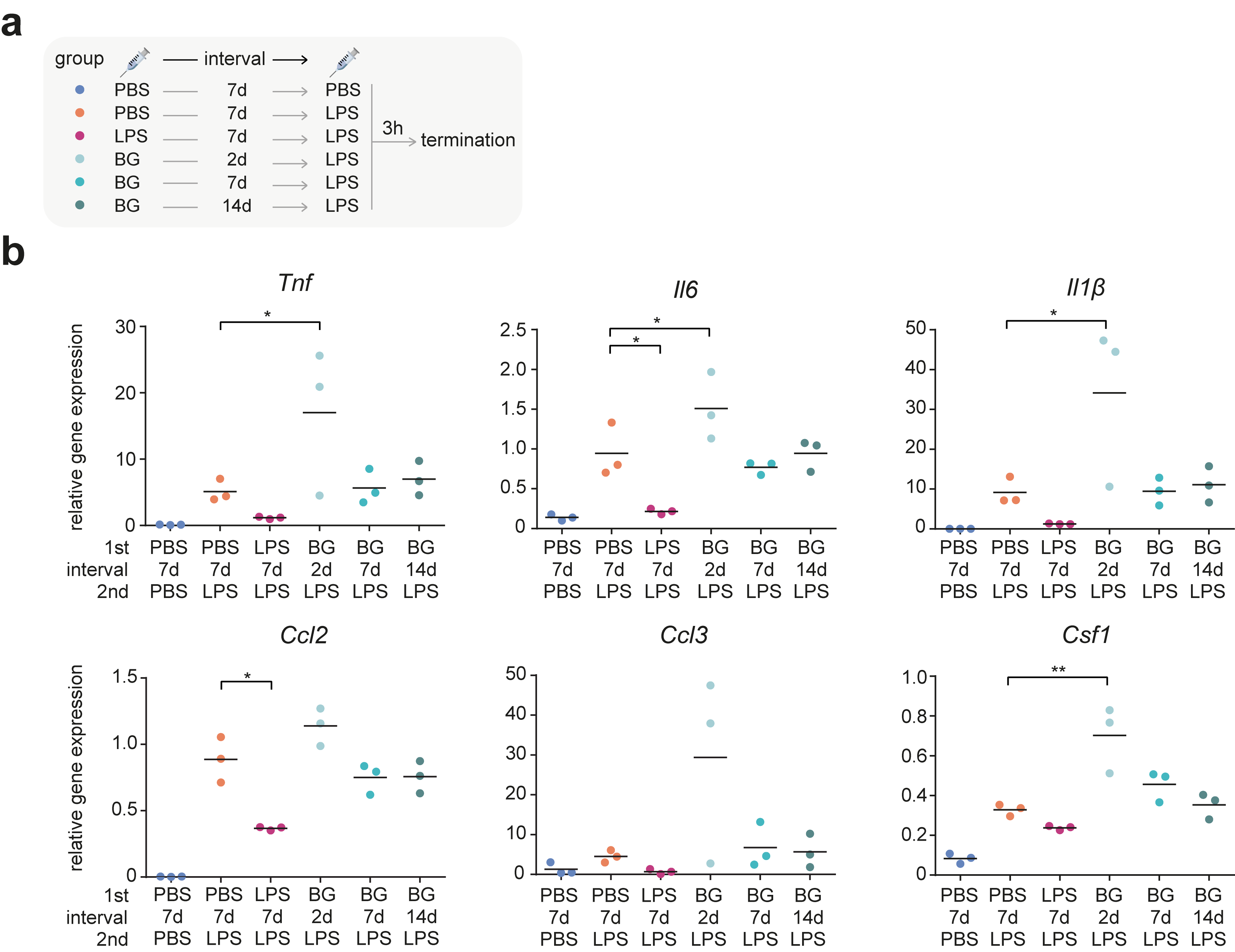

Supplement: Supplementary file 6 — Additional file 6 BG induces immune training in microglia in vivo 2 days after the first injection. a) Diagram of preconditioning experimental design. Mice received first challenge of PBS, LPS (1 mg/kg) or BG (20 mg/kg) by i.p. injection. After 2, 7 or 14 days, the same mice received second challenge with PBS or LPS (1 mg/kg). Animals were terminated 3 h after the second injection. b) Microglia were isolated and Tnf, Il6, Il-1β, Ccl2, Ccl3 and Csf1 gene expression levels were detected by qPCR and normalized to Hprt1 gene expression levels (n = 3 mice). [file 12974_2021_2103_MOESM6_ESM.tif]

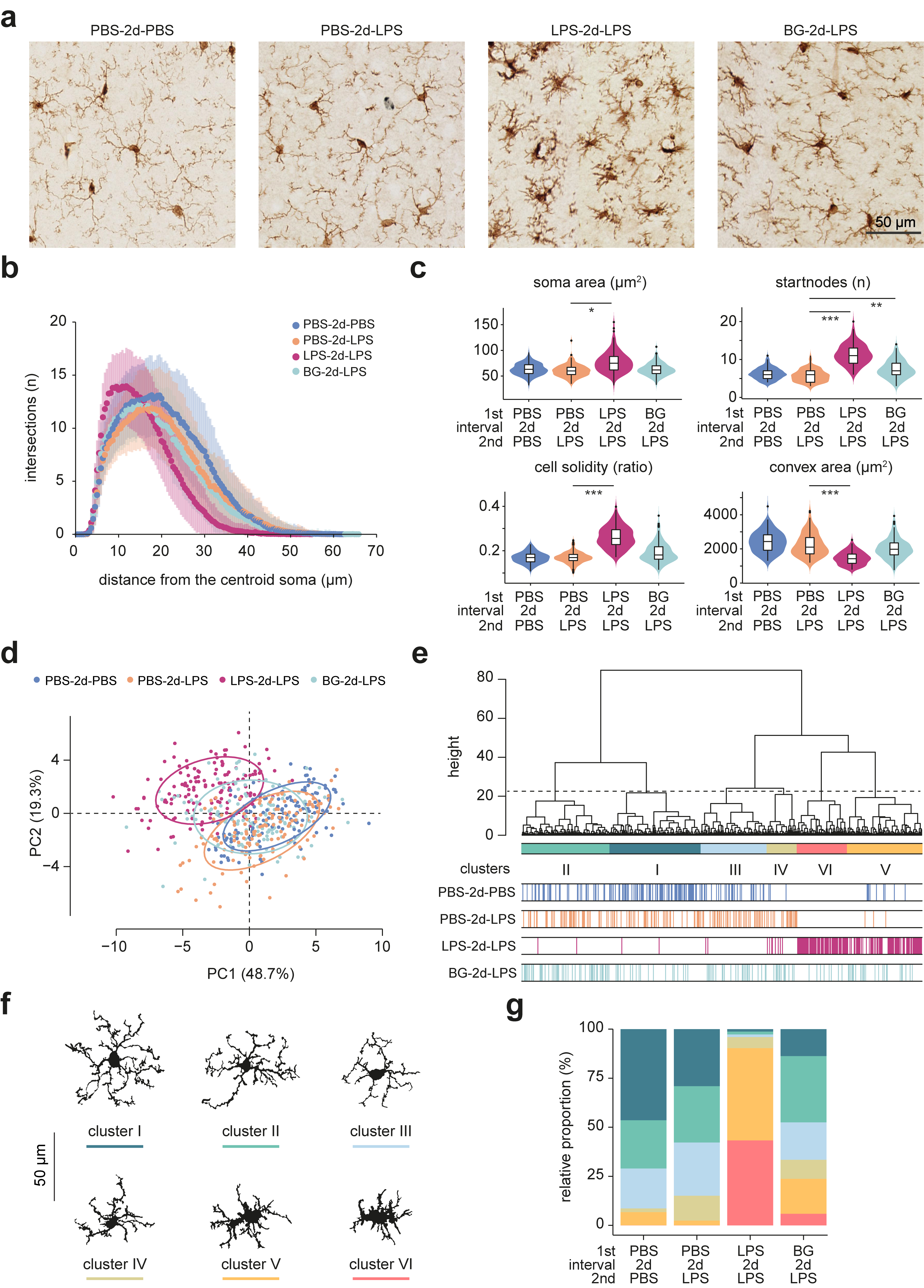

Supplement: Supplementary file 8 — Additional file 8. LPS- and BG-preconditioned mice microglia display an exaggerated reactive morphotype in response to a second LPS injection in hippocampus. a) Representative images of Iba1-staining in the hippocampus across different groups (scale bar: 50 μm). b) Violin plots of representative morphometric parameters for hippocampal microglia across experimental groups. The significance was determined by Wald chi-square test for linear mixed models. *, p < 0.05; **, p < 0.01; ***, p < 0.001. c) Sholl analysis of hippocampal microglia across groups. The vertical lines represent +/- standard deviations. d) PCA plot depicts all individual cells selected in hippocampus on principal component plane. The x and y axes represent the first and second principal components (PC1 and PC2), respectively. e) Hierarchical clustering on principal components resulted in 6 cell clusters (I-VI). The dashed line indicates the cut off for 6 clusters. f) Representative cells from each cluster are shown. g) Cluster distribution analysis of LPS- and BG-preconditioned mice microglia 3 h after second LPS challenge in hippocampus. Microglia from naïve mice with two PBS injections (PBS-2d-PBS group) and mice that only received one LPS challenge for 3 h (PBS-2d-LPS group) were included as controls. The number of microglia selected in the different groups: PBS-2d-PBS: 139 cells; PBS-2d-LPS: 138 cells; LPS-2d-LPS: 144 cells; BG-2d-LPS: 137 cells (n= 4 mice for each experimental group). [file 12974_2021_2103_MOESM8_ESM.tif]
